# Improving Haptic Response for Contextual Human Robot Interaction

**DOI:** 10.3390/s22052040

**Published:** 2022-03-05

**Authors:** Stanley Mugisha, Vamsi Krisha Guda, Christine Chevallereau, Matteo Zoppi, Rezia Molfino, Damien Chablat

**Affiliations:** 1 Dipartimento di Ingegneria Meccanica, Energetica, Gestionale e dei Trasporti, University of Genova, Via All’Opera Pia, 15, 16145 Genova, Italy; matteo.zoppi@unige.it (M.Z.); rezia.molfino@unige.it (R.M.); 2 CNRS, LS2N, UMR 6004, 1 Rue de la Noë, 44321 Nantes, France; vamsikrishna.guda@ls2n.fr (V.K.G.); christine.chevallereau@ls2n.fr (C.C.); damien.chablat@cnrs.fr (D.C.)

**Keywords:** haptic devices, response time, human–robot interaction, virtual reality, eye–gaze tracking

## Abstract

For haptic interaction, a user in a virtual environment needs to interact with proxies attached to a robot. The device must be at the exact location defined in the virtual environment in time. However, due to device limitations, delays are always unavoidable. One of the solutions to improve the device response is to infer human intended motion and move the robot at the earliest time possible to the desired goal. This paper presents an experimental study to improve the prediction time and reduce the robot time taken to reach the desired position. We developed motion strategies based on the hand motion and eye-gaze direction to determine the point of user interaction in a virtual environment. To assess the performance of the strategies, we conducted a subject-based experiment using an exergame for reach and grab tasks designed for upper limb rehabilitation training. The experimental results in this study revealed that eye-gaze-based prediction significantly improved the detection time by 37% and the robot time taken to reach the target by 27%. Further analysis provided more insight on the effect of the eye-gaze window and the hand threshold on the device response for the experimental task.

## 1. Introduction

Haptic systems enable user interaction in virtual reality by automatically recreating virtual scenes for dynamic interactions through haptic rendering, thus creating a link between a virtual world and the real world. Haptic systems should allow for a wide range of physical interactions and manipulations throughout the user’s workspace, with a physical input that resembles reality. One promising approach to achieve this is the paradigm of encountered-type haptics (EHDs) [1]. EHDs are devices that autonomously position physical props for virtual objects in the real world at a target appropriately, thus allowing users to reach out to the virtual objects physically, just like in the real world. However, it is challenging for real-time interaction to organize physical props that accurately replicate the virtual world due to practical constraints, such as speed and workspace limits. In addition, the virtual environments are always much more extensive and richer in variety than the tracked physical space [2]. Speed limitations delay the device’s arrival to some targets, creating discrepancies between what the user can see and what they feel. The resulting position and orientation mismatch between the virtual object and haptic proxy and latency negatively impacts the user experience [3,4]. While these issues may be partly solved by improving device hardware, factors such as cost, safety, and complexity often lead to design decisions that make device workspace and speed constraints unavoidable. Control approaches from the state-of-the-art, such as haptic-retargeting [2] and user motion prediction, have been employed to address speed and latency issues [5]. Our study addresses this problem through motion prediction using the human eye-gaze tracking and hand motion. Previous studies have shown that the head movement facilitates subsequent gaze shifts toward the future position of the hand to guide object manipulations [6,7]. Thus, tracking eye movements is a natural way to learn about an intended reach target [8]. With eye-gaze information, hand movements, and the information in virtual environment, we can predict the tasks that the user will perform. Eye-tracking systems have been found to play an increasingly important role in assistive robotics as hand-free interaction interfaces for motor-impaired people [9], social gaze control for humanoids [10], robotic guidance [11], creating artistic drawings [12], and safe robot interactions in patients with speech and motor impairments [13]. Eye-tracking combined with action observation tasks in a virtual reality display has been used to monitor motor deficits derived from stroke and, consequently, for the rehabilitation of stroke patients [9,14].

This study aims to develop and evaluate motion prediction strategies by analyzing the hand motion and eye gaze of adults when selecting targets. The strategies are used for upper limb training exercises to simulate activities of daily living tasks for people with motor impairments.

The main contributions of this work are:

1. We introduce and compare three strategies to detect human intention using the eye gaze and the hand motion to improve the human immersion.We use the eye-gaze detection rather than the eye-gaze attention used in [2,15,16];
2. We introduce a framework to implement the strategies;
3. We implement a proof of concept that illustrates our proposed approach;
4. We study the effect of the eye-gaze field of view and the threshold by comparing our approach to state-of-the-art eye-gaze-based robot control.

The remaining part of the paper is structured as follows. Section 2 discusses work related to haptic displays and prediction strategies. Section 3 describes the context of the study, the intention prediction strategies, the design and setup of the human–robot interaction model to contextualize the contribution of this research, the evaluation criteria, and the experimental design. Section 4 presents the results of the analysis of the performance of the strategies, and Section 5 discusses the results.

## 2. Related Works

This section focuses on haptic display devices, and the options researchers seek to improve surface rendering. Then, the state-of-the-art on human intention detection through motion predicting algorithms is presented.

### 2.1. Haptic Displays

There is a significant amount of studies on haptic devices in the literature. Our review will focus on encounter-type haptics, which employ a prop attached to a robot. The earliest work, Mcneely [17], presented the concept of encountered-type haptic device. The system places a haptic device at the desired location in time and waits for the user’s encounter. It has the extra benefit of allowing the user’s hand to move freely in open space and the use of physical props attached to a robot to represent virtual objects with varying sizes and shapes [18,19]. Other devices followed, such as the shape approximation device [20], haptic simulation of the refrigerator door [21], and a robotic turret with switches [22]. Surface rendering with texture and temperature characteristics [23] and new forms of EHDs, including shape-changing displays [24], surrounding platforms [25], mobile robots [26,27], and drones [28,29] also followed. To enable smooth interactions, EHDs need to achieve a high level of spatial–temporal consistency between the visual and haptic sensory inputs [1]. However, EHDs have limitations that lead to discrepancies between what the user can see and what can be felt, including limited workspace volumes, positional inaccuracy, and low speeds that may not support real-time interactions.

### 2.2. User Motion Prediction

Motion prediction strategies to determine the next target the human would like to reach and action to take can overcome timing constraints that affect most EHDs. This section explores the prediction and intention detection in the literature. Mostly, machine learning techniques, such as neural networks [30,31,32], Bayesian methods [32,33], principal component analysis [34], dynamic movement primitive [35], and hidden Markov models [36], have been used. A probabilistic principal component analysis was used for the recognition and prediction of human motion through motion onset detection by relying on a motion detection database of various motion models and an estimation of the execution speed of a motion [34]. Li et al. used a Bayesian predictor for the motion trajectory of the human arm in a reaching task by combining early partial trajectory classification and human motion regression in addition to neural networks used to model the non-linearity and uncertainty of human hand motion [32]. A combination of hidden Markov models and probability density functions was used in [36] to model the human arm motion and predict regions of the workspace occupied by the human using a 3D camera. In a related work, a Bayesian inference model [33] was used to infer the hand target and to promptly allow for the robot to reach a position within the scene. Based on observations from a 3D camera sensor, Ravichandar et al. [30] trained a neural network using a data set containing demonstrations of a human reaching for predefined target locations in a given workspace to infer a goal location for the human hand reach. However, all of the above models require vast amounts of training data. Furthermore, the performance of the models is dependent on the data acquired. Therefore the performance is affected when new measurements are received due to arm motion dynamics or different conditions of the human subjects. Other techniques that do not use training data are based on a distance metric [37]. This method selects an object closest to the user’s hand by calculating the distance of all objects of interest in the scene from the hand and selecting the best. However, this method only detects the next desired object only when the hand has crossed the midpoint or has gone beyond the current minimum distance; therefore, if two objects are far apart, detecting the next one will take a longer time.

Since the hand position is one of the most informative features in human manipulation movement, the above works on intention inference based on hand motion. However, based on assumptions from studies on human behaviour, for most tasks involving object manipulation, humans reach to grasp an object and look at the target first. The gaze direction is always in the direction of the hands and the object manipulated [6,7], and therefore can be used to determine targets for interaction.

The eyes are considered as a window into the human mind because they can reveal information about human thoughts and intentions, as well as our emotional and mental states and where we are paying attention to [38]. Thus, the eye gaze can be used as a direct input to control robots and predict users’ targets. Gonzalez et al. [2] used gaze fixation to predict an element of a virtual scene the user wants to reach. If the robot could not arrive at that target in time, they remapped the virtual element to a physical point within the EHD’s reachable space. Stolzenwald et al. [15] introduced a model that predicts users’ interaction location targets based on their eye gaze and task states using a hand-held robot. This model derives intention from the combined information about the user’s gaze pattern and task knowledge. Castellanos et al. used eye-gaze information to predict the user target and provided haptic assistance for people with physical disabilities [16]. These works use gaze fixation to select the desired target. To classify an object as the target, they wait for a time ranging from 200 ms to 4 s when the eyes are fixated on an object. However, this approach results in unnecessary delays and may not be practical for smaller objects.

Using additional data from the head-mounted display, we use the gaze direction and only consider the points in the user-facing direction. The desired point is selected from a few candidate points within a defined threshold distance from the hand and a user view cone. In this approach, points that were not in the gaze direction or above the threshold were not considered, even if they were close to the user’s hand. Our approach aims to pre-select all objects the user views and then select the desired object in the eye-gaze direction. Our method is designed to work with hand motions during real-world interaction and to give participants the freedom to make their own decisions along the way.

## 3. Methods

### 3.1. Context of Study

**Figure 1:**
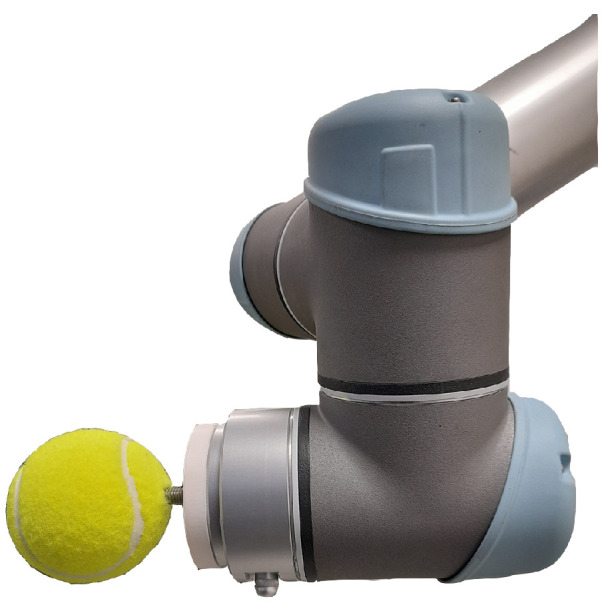
The designed prop, a physical representation of the virtual objects presented to the user during interaction.

**Figure 2:**
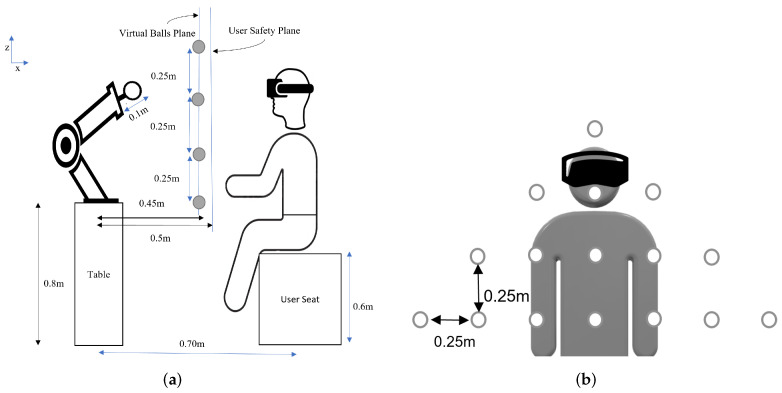
Experimental setup with the robot, the balls, and the user. (**a**) The side view. (**b**) The front view.

We based this study on an exergame designed for upper limb rehabilitation training for both the right and the left hand. The task aims to simulate reaching and grabbing balls in a virtual space. The study is inspired by the work presented in [39] for upper-limb and postural rehabilitation. Different balls are displayed to the player at different locations at a given time instance. He/she has to reach and grasp a ball of choice and release it above the virtual basket on the floor to gain points. The exergame is designed to record the active range of the motion of the user’s hand using HTC Vive trackers. Then, the data are used by a control algorithm to generate virtual objects within the patient’s comfort zone initially, and then to gradually push them further out of the comfort zone. The virtual world application allows the user to perform daily life activities while providing abundant repetitive movements and giving the patient visual feedback. The game was developed in collaboration with researchers and physiotherapists at the University of Genoa and LS2N. In this scenario, the user sits in the real world on a chair for a visual virtual reality experience and must reach out to pick balls with one of his or her hands. While the user is attempting to interact with a virtual object in the environment, the robot must position a ball to provide a tactical sense of touching the object [18,19]. A motion capture system based on HTC Vive trackers is used to determine the position of the hand used for interaction and the position of the chair and the robot. A tennis ball was attached to the robot’s flange, as shown in Figure 1. The robot was mounted on a 0.8 m high table. The user was seated on a seat, positioned 0.6 m above the floor and 0.7 m from the robot. The robot’s placement in the scene was chosen in order to allow it to reach all of the locations where the user’s hand will want to have a haptic interaction with the robot’s prop, as shown in Figure 2a,b. The arrangement of the balls fixed in the environment is represented by a virtual model created by the Unity© software.

The main components for this study were:

1. An encountered-type haptic device comprising a grounded Universal Robots UR5 robotic arm. A spherical prop was attached to give the sensation of touching a ball using a dominant hand;
2. A motion capture system. The HTC Vive pro eye VR headset/head-mounted display for eye-tracking, a Vive tracker and base stations for tracking users’ hand position, and another Vive tracker at the robot’s base for robot positioning;
3. Virtual Environment: Virtual objects were rendered using the Unity software along with the intention detection strategies. The Tobii XR SDK (Tobii Technology Inc., Stockholm, Sweden) captured and processed gaze data;
4. Motion planning and obstacle avoidance. The algorithms for collision-free path and execution of the desired trajectories were implemented in ROS by using MoveIt function [40]. In the implementation, we ensured that the new objective is defined only when the robot has stopped. To avoid the computation of collision-free path, all trajectories used were pre-computed, and no new trajectory was generated during the experiments. The details of the implementation of the trajectory planning, collision, and obstacle avoidance algorithms are explained in [41].

### 3.2. Detection Strategies

Since the user has many balls presented in a virtual 3D environment at a given time instance, they have to choose one at a time. The robot’s task is to arrive at the desired position in time. Different strategies were proposed to read the intention of the human in order to predict which ball he/she may want to reach.

#### 3.2.1. Strategy 1: The Nearest Neighbour Approach

**Figure 3:**
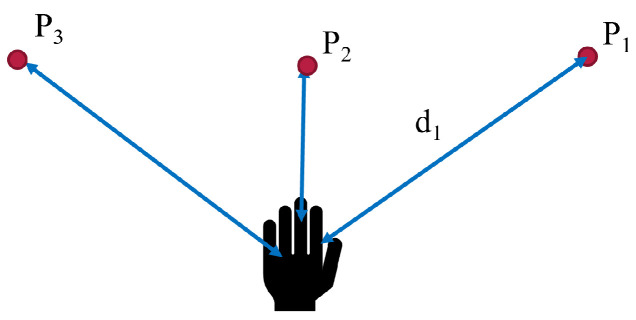
Pictorial representation of strategy 1.

The most commonly used approach depends on finding the object closest to the hand. Implementation was carried out by computing the distances from the hand to all points of interest in search space, as used in [37], or, alternatively, by searching through a k-d tree, as used in [5]. In this study, we used a k-d tree to store the positions of all objects in the scene. Using hand location based on data from the tracker, we searched for the nearest to the hand from the k-d tree using Algorithm 1, as shown in Figure 3. The desired point is the closest to the hand, corresponding to $\min(d_{i},d_{N})$: in this case, $P_{2}$.

However, the main drawback is that the target is detected only when the human hand has almost reached the point. Moreover, if two points are close, switching between two points can occur.
**Algorithm 1** Strategy 1: Predictions with hand.**Input:** Hand position $P_{h}\in R^{3}$.**Output:** Best point P∗ in the set of $P_{i}$, i=1…16. 1: Build a k-d tree for all points $P_{i}$ in the scene. 2: **function** ST1($P_{h}$) 3:     Using hand pose as a query point *q*, return nearest point from the k-d tree. 4:     **return** P∗ 5: **end function**

#### 3.2.2. Strategy 2: Hand Position with Threshold

**Figure 4:**
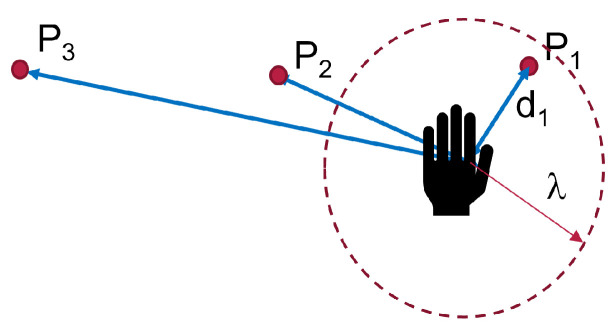
Pictorial representation of strategy 2.

To detect the next desired point, a threshold distance between the hand and the current point of interaction was used to detect if the user intends to move their hand or if their hand is close to a point. Once the distance between the hand and the current point is above the threshold, we maintained the previous, and if the next point the hand approaches is within the threshold, it was taken as the indented target. The threshold ensures that only points in close contact are selected as explained in Algorithm 2. In this way, we aimed to reduce the detection of intermediate points and, hence, reduce the number of erroneous points detected. In Figure 4, the best point would be $P_{1}$.
**Algorithm 2** Strategy 2: Hand position with threshold.**Input:** Hand position $P_{h}\in R^{3}$, threshold distance $\lambda_{d}$.**Output:** Best point P∗ in the set of $P_{i}$, i=1…16. 1: Build a k-d tree for all points $P_{i}$ in the scene. 2: **function** ST2($P_{h}$, $P_{prev}$) 3:     $P_{next}$← best from k-d tree. 4:     **if** $\left|\right|\left(P_{next},P_{h}\right)\left|\right|<\lambda_{d}$ **then** 5:         $P*\leftarrow P_{next}$ 6:     **else** 7:         $P*\leftarrow P_{prev}$ 8:     **end if** 9:     $P_{prev}\leftarrow P*$
 10:    **return** P  11: **end function**

#### 3.2.3. Strategy 3: Using Eye Gaze, Prediction with Eye Gaze

In addition to the hand position, strategy 3 uses eye-gaze direction to determine the next point. The next target is the point closest to the ray from the midpoint of the eyes in the gaze direction. As in previous works, the difference with this approach is that we do not wait for the gaze fixation on a specific object. In this approach, the detection is guaranteed to be fast. A threshold distance $\lambda_{d}$ was added onto the hand to detect when the user intends to move. If the hand to the point distance is within a threshold, we assumed that the user is still interacting with the current point. The value of $\lambda_{d}$ was chosen so that only one point $P_{i}$ can be inside. However, if the distance is above the threshold, the human wants to move to the next point; therefore, a new target was selected based on the eye-gaze direction. In this case, the threshold serves two roles. The first is to detect the intention of the user to move and then to cut off the selection of the next point by the head gaze. The threshold stops the robot from moving when the hand is near a point.

The search by gaze direction starts with only the points in the view frustum of the HMD. We carried this out to limit the search space and improve the detection speed.

In addition, we added a limit α on the angle from the gaze line to restrict the points selected by the eye gaze. The angle can be varied from 1%, as was used in [2] for a visual attention task. Another study [42] on visual attention perspective for social robotics modeled the threshold as a cone model of $30^{\circ }$, whereas [43] used a slightly wider aperture of $40^{\circ }$.

**Figure 5:**
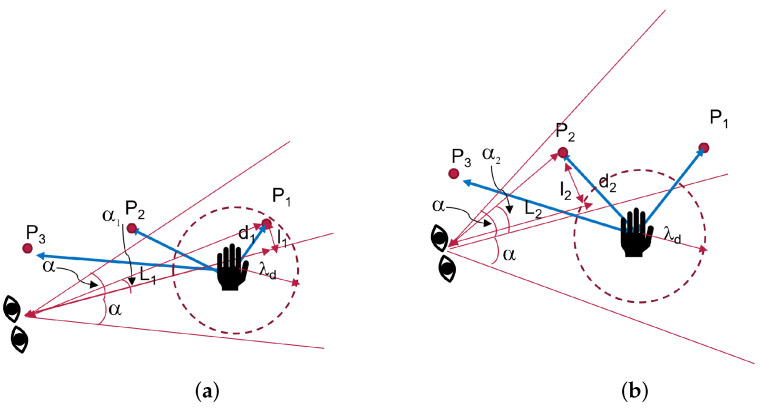
Strategy 3 prediction with eye gaze tracking. (**a**) Case 1: Point within $\lambda_{d}$, (**b**) Case 2: All points outside $\lambda_{d}$.

If there is no point within the limit α, the previous point was maintained. The ray in the gaze direction was then used to determine the next target. If the point-to-hand distance is above the threshold $\lambda_{d}$, the point selected by the head gaze was taken as the desired target. Otherwise, it was ignored, and robot motion was restricted to the point near the hand. As shown in Figure 5a, the best point selected is $P_{1}$.

We started by building a list of all points in the user view and then calculated the angle $\alpha_{i}$ using Equation (1) for each point $P_{i}\in P$. The next target is the point with a minimal $\alpha_{i}<\alpha$ value.

(1) $$\alpha_{i}=\tan^{-1}\left(\frac{l_{i}}{L_{i}}\right)$$

$l_{i}$ is the projection of a point $P_{i}$ on the ray in the gaze direction and $L_{i}$ is the distance of the projection point to the center of the eyes. Algorithm 3 describes the procedure in two steps:

1. Case 1: The hand is very close to a point as in Figure 5a. Search for the best $P_{i}$ using the strategy 1
  If $\left|\right|\left(P_{i},P_{h}\right)\left|\right|<\lambda_{d}$, then $P^{*}\leftarrow P_{i}$;
2. Case 2: All points are very far as in Figure 5b. Next point is determined by eye gaze. $P^{*}\leftarrow P_{next}$ from Equation (1).

**Algorithm 3** Strategy 3: Predictions with head gaze and threshold on eye-gaze angle.**Input:** Hand position $P_{h}\in R^{3}$, Gaze direction vector $G_{d}\in R^{3}$, hand threshold $\lambda_{d}$, head gaze threshold α.**Output:** Best point P∗ in the set of $P_{i}$, i=1…16. **function** ST3($P_{h},G_{d},P_{prev}$)     $p_{bh}\leftarrow$ best point position from the KD tree. 3:     **if** ($\left||(\left(P_{bh},P_{h}\right)||<\lambda_{d}\right)$ **then**           $P*\leftarrow P_{bh}$      **else** 6:         build a list of points *P* in the view frustum of HMD with $\alpha_{i}<\alpha$.           **if** P=∅ **then**              $P*\leftarrow P_{prev}$ 9:         **else**              $p_{next}\leftarrow$ point with $\min(\alpha_{i})$ from the list *P*.              $P*\leftarrow P_{next}$ 12:         **end if**      **end if**      $P_{prev}\leftarrow P*$ 15: **end function**

### 3.3. Data Flow and System Integration

**Figure 6:**
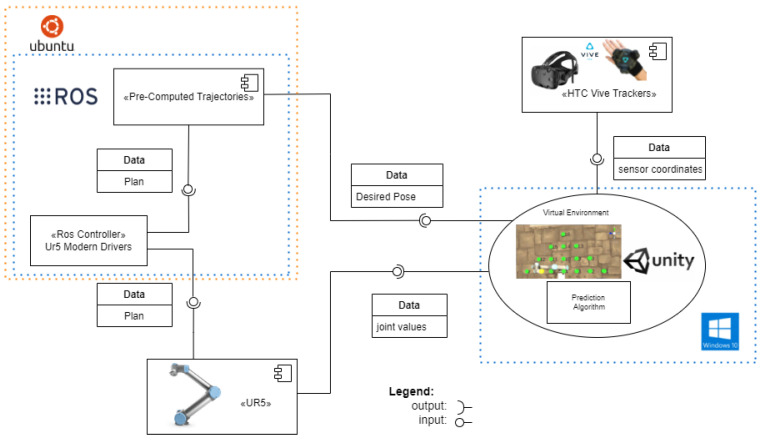
Flowchart of software and hardware used.

The data exchange for the above system components is shown in Figure 6. The proposed architecture describes the different interactions each system element has and provides an insight into how the instances share the information and communicate to each other.

The ROS component receives just the desired goal as an input. Later, based on this information, the move_group can generate a plan for the robot to reach the desired positions using pre-computed trajectories. Once the plan is generated, we communicated to the UR5 robot by using the “ur_modern_driver” [44]. With it, we can move the UR5 robot with ROS control and send, as an output, the current joint states of the robot for the Unity system to work with.

### 3.4. Experimental Setup

The UR5 Universal Robot was used to implement the system. This robot was programmed to receive a desired position and orientation from Unity software and move the prop. Participants used their right hand to touch the prop. For the training, the HTC Vive tracking system was set up in a room without external disturbance, and the user was positioned at a distance of 0.7 m from the robot. A tracker was attached to the user’s hand for motion capture in 3D space for interaction within game activities. The user held no other devices.

To ensure safety of the user, the workspace was divided by a safety plane into the human workspace and the robot workspace. The safety plane was used to restrict the motion of the robot to the robot workspace by using a motion planning algorithm described in [41]. In this study, the plane was considered as a static obstacle to be avoided. In addition, there was an emergency switch making it possible to cut off power to the whole system by flicking a switch.

### 3.5. Experimental Task

**Figure 7:**
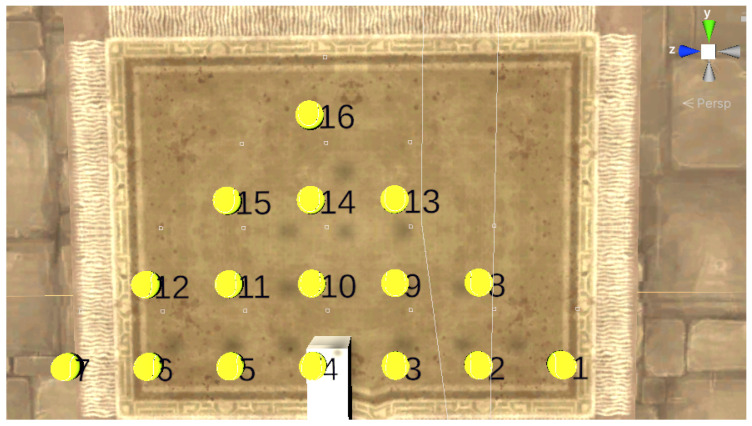
Virtual environment rendering of the scene.

The task comprised 16 tennis balls displayed in a virtual environment, located at points $P_{1}$ to $P_{16}$, and spawned within the robot workspace, as shown in Figure 7. Three volunteers participated in this experiment. They included 1 female and 2 male participants with a mean age of 32 years. None of them had experience with eye-tracking displays; however, 1 of them had used a VR display. All participants were right-handed and provided written informed consent prior to the start of the experiment. Each participant was told to move the dominant hand from a ball specified by a number to a target ball also specified by a number.

**Figure 8:**
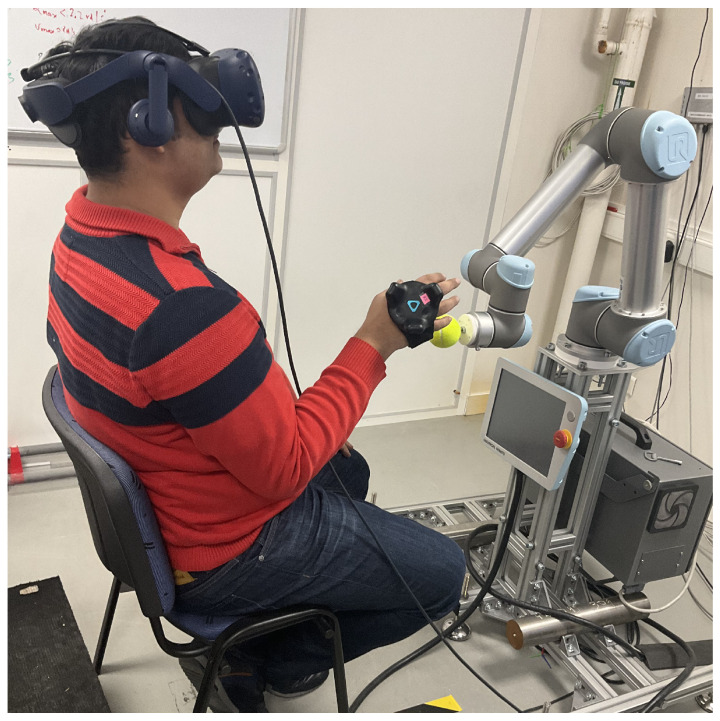
The user performing the experimental task in unity without motion of the robot.

The participants performed the task of reaching toward and grasping a ball with a radius of 7 cm and matching 3D virtual renderings as shown in Figure 8. The physical object was 3D-printed thermoplastic. Participants wore a head-mounted display to provide a 90 Hz virtual picture update frequency and scene sound effects while a tracker was attached to the hand. They viewed green-colored virtual renderings of these objects and a virtual rendering of the hand in a custom 3D immersive virtual environment designed in UNITY (ver. 19.4.1f1, Unity Technologies, San Francisco, CA, USA). The objects in the virtual environment were placed at different locations corresponding to the length of the arm 1/3 length of the arm at 25 cm from the centre (near), 2/3 arm length (middle) at 50 cm from the centre, and full arm length at 75 cm from the centre (far), each corresponding to a level of difficulty. A computer with an Intel Core i7-7700 processor and an NVIDIA gtx 2070 graphics processor was used to create the virtual environment.

### 3.6. Design of the Experiment

The main objective was to study the effect of the eye gaze on the detection time of the desired point, intermediate points, the time taken by the robot to reach the desired point, and intermediate stops of the robot. For this, three strategies were compared. The nearest-neighbour method [5,37] (strategy 1) was used as the baseline. The null hypothesis was that eye-gaze-based prediction had similar results as the selection with the hand-alone strategies for the detection time, intermediate points detected, robot time, and intermediate points for the robot. For the research objective, the following evaluation criteria were defined:

- Q1: The time taken by the strategy to detect the desired point;
- Q2: The number of intermediate points detected by the strategy before the desired point was detected;
- Q3: The time taken by the robot to reach the final point. This was the sum of the duration of all of the pre-computed trajectories plus the waiting time of the robot;
- Q4: The total number of intermediate stopping points of the robot.

### 3.7. Data Collection

We recorded the participant’s hand position, head position, and eye-gaze direction for each point-to-point trajectory. Data for the following trajectories were recorded:

- Long trajectories: $P_{1}-P_{6}$, $P_{1}-P_{16}$, $P_{7}-P_{16}$, $P_{1}-P_{7}$ and $P_{4}-P_{16}$;
- Medium trajectories: $P_{1}-P_{13}$, $P_{7}-P_{15}$, $P_{12}-P_{13}$ and $P_{8}-P_{12}$;
- Short trajectories: $P_{6}-P_{14}$, $P_{6}-P_{9}$, $P_{3}-P_{11}$, $P_{13}-P_{16}$ and $P_{14}-P_{16}$.

## 4. Results

Out of the 39 recorded trajectories, one was discarded due to recording errors, and the remaining 38 were used for analysis. We first present a detailed analysis of an individual trajectory, then a summary of the results from 38 trajectories on Q1, Q2, Q3, and Q4, then an analysis on the effect of the hand threshold, and finally the effect of the eye-gaze window. It is important to note that, for the analysis of the results, the values of $\lambda_{d}$ = 0.15 m and the value on the eye-gaze threshold in strategy 3 was $60^{\circ }$.

### 4.1. Analysis of the Trajectory from $P_{1}$ to $P_{16}$

**Figure 9:**
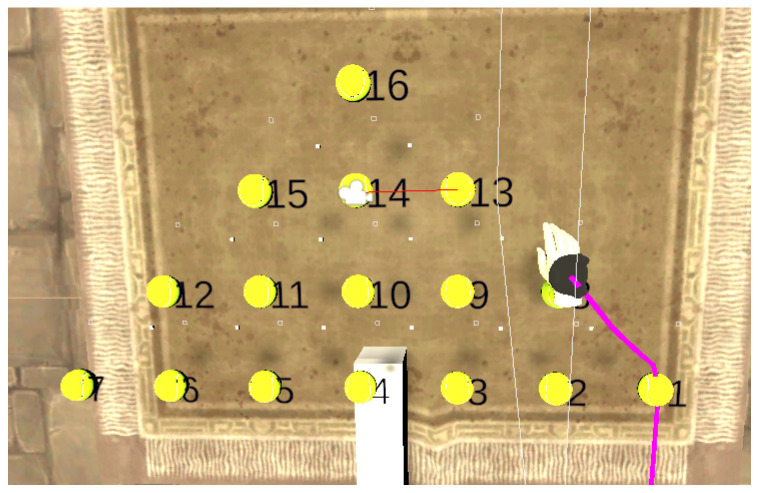
User’s hand trail and selection by eye gaze for motion from $P_{1}$ to $P_{16}$. The hand trail is shown as a pink line. The ball selected by the eye-gaze direction is $P_{13}$, indicated by a slim red line from the camera, represented as an icon.

**Figure 10:**
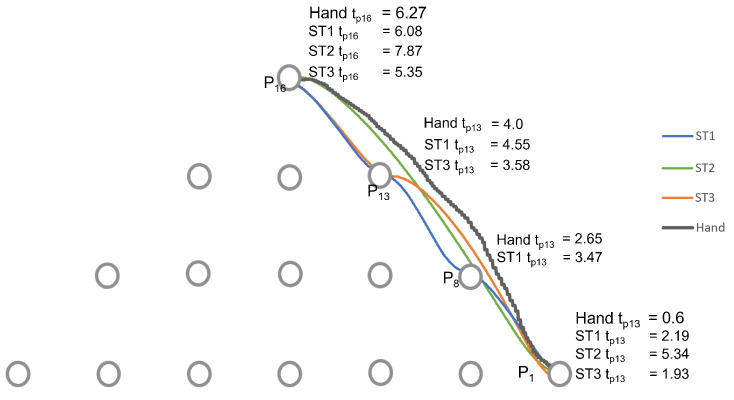
A representation of the actual hand motion and the resultant robot motion projected on the y-z plane. The time the robot stops at a point is indicated for each strategy, as well as the time the hand is closest to each point. For $p_{1}$, the time at which the motion starts is indicated. For $p_{8}$, $p_{13}$, and $p_{16}$, the time the robot stops is indicated. For $p_{16}$, the time the hand stops is indicated. For $p_{8}$ and $p_{13}$, the hand that is the closest is indicated.

**Figure 11:**
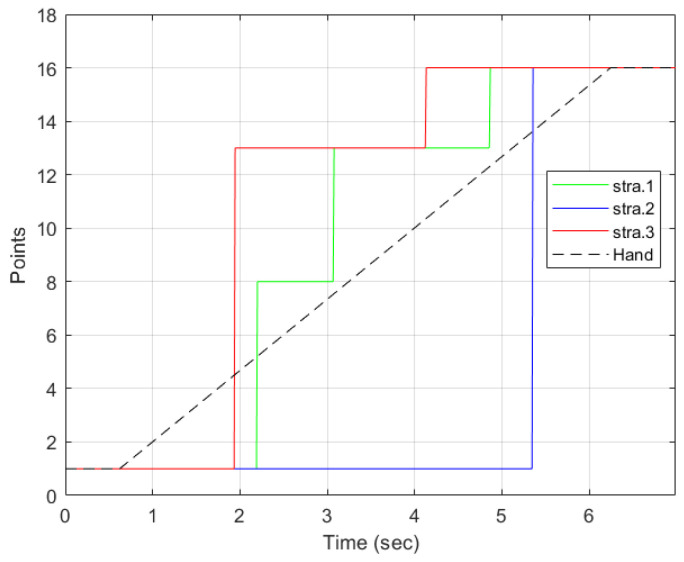
Comparison of the strategies for the trajectory from $P_{1}$ to $P_{16}$. The dotted line indicates the time the hand is at the start and the target point.

We took, as an example, one of the user’s motion trajectory from point $P_{1}$ to $P_{16}$ to analyze the results of the three strategies proposed based on the four criteria Q1, Q2, Q3, and Q4 (as shown in Table 1). A user view is shown in Figure 9 using strategy 3. The robot motion corresponding to each strategy is shown in Figure 10 and Figure 11. In Figure 10, we represent the hand trajectory and the resultant robot motion for the different strategies. For $P_{1}$, we only show when the motion started. For the rest of the points, we indicate the time at which the hand was closest to each point, and the time the robot stopped at any point. A video of a user performing a motion from point $P_{1}$ to $P_{10}$ and $P_{1}$ to $P_{16}$ using strategy 3 along with the robot is provided in the Supplementary Materials.

#### 4.1.1. Strategy 1

**Figure 12:**
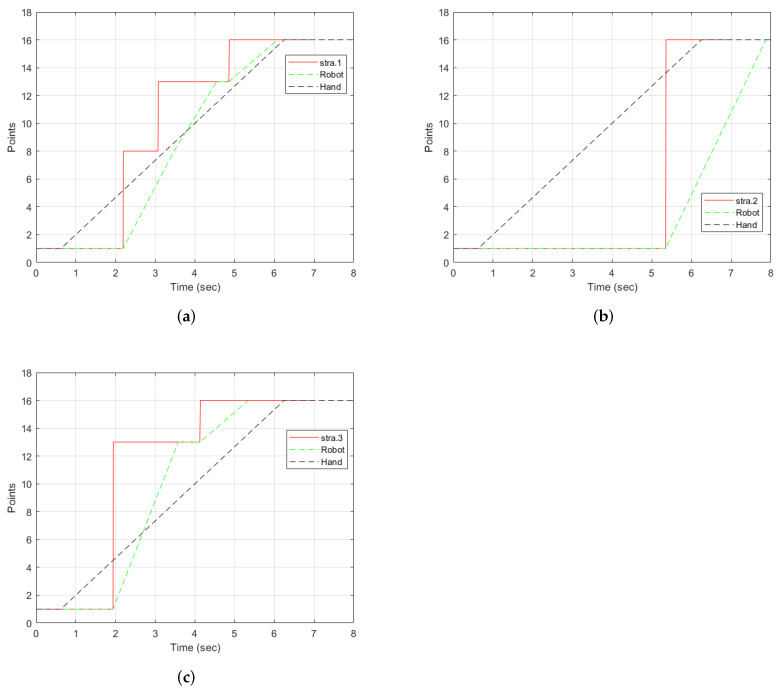
Results of individual strategies selection with the hand and robot motion for the trajectory from $P_{1}$ to $P_{16}$ showing the points selected by the strategies, the robot, and the hand stops. The dotted lines represent the motion of the hand and the robot. The graphs only show the points and the time the hand and robot stop. (**a**) Selected points using strategy 1 along with the robot and hand stops. (**b**) Strategy 2 points selection along with the robot and hand stops. (**c**) Strategy 3 selection along with the robot and hand stops.

With strategy 1 as illustrated in Figure 12a, the points detected were $P_{1}$, $P_{8}$, $P_{13}$, and $P_{16}$. The desired point was detected at t=4.86 s. Two intermediate points were detected: $P_{8}$ and $P_{13}$. Points 8 and 13 are along the path of the straight line. Therefore, each of them was detected as the hand moved. The robot stopped at all the points detected, as indicated by the green line. The hand left from $P_{1}$ at t=0.6 s. $P_{8}$ was the first point to be detected by the strategy and the robot received the point and moved towards it. However, before reaching $P_{8}$, the strategy detected $P_{13}$. Since the trajectory from $P_{1}$ to $P_{8}$ was not yet completed, the robot reached $P_{8}$, stopped, and then started a new trajectory from $P_{8}$ to $P_{13}$. It then waited for new information to go to $P_{16}$.

#### 4.1.2. Strategy 2

The motion of the hand, the robot, and the selection by strategy 2 is shown in Figure 12b. From $P_{1}$, the strategy selected $P_{16}$ at t=5.34 s. There were no intermediate points detected. This was possible because the hand threshold limits the selection of a point until the condition is met. This can be an advantage if the objective is to minimize the detection of unwanted points. However, it comes with a cost of late detection of the desired point when compared to other strategies, as shown in Figure 11. The robot moves directly to the desired point. However, it arrives after the hand has already reached the point.

#### 4.1.3. Strategy 3

Figure 12c illustrates the progression of strategy 3. The strategy started with point $P_{1}$, then selected $P_{13}$, the best point in the user eye-gaze direction shown in Figure 9 by the red line on the camera icon, and, then, finally, $P_{16}$. The robot started from point $P_{1}$, then to the intermediate point $P_{13}$, where it waited for a new point, and then to $P_{16}$. As can be observed in the graph, the robot arrived at the final point earlier than the hand and the other strategies.

### 4.2. Analysis for All Trajectories

For all of the objectives Q1, Q2, Q3, and Q4, the data distribution was checked for normality using the Shapiro–Wilk test [45]. We used the strategies as a three-level factor and strategy 1 as the baseline for comparison. A one-way analysis of variance (ANOVA) model was used to fit the data. Results showed that there were significant differences among the strategies (p<0.05) for all the objectives, with Q1 (F(2,111)=10.66 and p=0.000), Q2 (F(2,111)=19.21 and p=0.000), Q3 (F(2,111)=10.77 and p=0.0), and Q4 (F(2,111)=30.62 and p=0.000). Therefore, we reject the null hypothesis and conclude that the mean detection time, the number of intermediate points detected, robot arrival time, and the intermediate points detected by the robot are different for all the strategies. The results indicated that the effect of the eye-gaze tracking was significant for all of the objectives. A post hoc analysis was performed to find out the strategy-wise differences using the Bonferroni [46] and the Tukey test [47].

**Figure 13:**
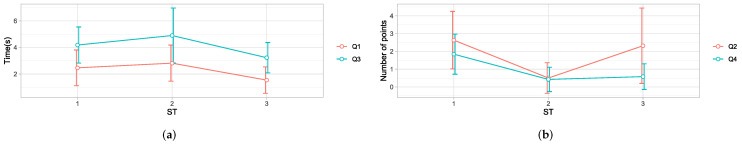
Results for each strategy. (**a**) Q1: Time taken for each strategy to detect the desired point. Q3: Time taken by the robot to reach the desired point. (**b**) Q2: The number of intermediate points detected by each strategy. Q4: The number of intermediate stopping points of the robot.

The Tukey test showed that the time for detection in strategy 3 was significantly lower than strategy 1 (p=0.004) and strategy 2 (p=0.000). Overall, strategy 3 was the best with the lowest time, as shown in Table 2 and Figure 13a. Compared to the baseline, the time difference was 0.92 s, representing a 37% reduction. However, there were no significant differences between the other strategies. These results indicate that the participants always looked in the direction of the desired point before moving their hand. Mutasim et al. [48] discovered similar results in a study of gaze movements in a VR hand-eye coordination training system. They found that the target was detected, on average, 250 ms before touch with eye gaze. Therefore, the use of eye-gaze direction tracking significantly reduced the detection time.

A post hoc analysis using the Tukey test showed that strategy 2 had a significantly reduced number of intermediate points detected compared to strategy 1 (p=0.000). The results can be seen in Table 2 and Figure 13b. The difference between strategy 3 and strategy 1 was insignificant, although strategy 3 had a lower number of intermediate points by 20%. Due to the rapid eye movements (the saccades), eye-gaze direction tracking can result in the detection of intermediate points. However, the hand threshold prevented the selection of a new target when the hand was close to a point, hence reducing the number of intermediate points in strategy 3.

Concerning the robot time, a post hoc analysis showed that the overall time taken for strategy 3 was significantly lower than strategy 1 (p=0.025) by 23% and strategy 2 (p=0.000), as shown in Table 2 and Figure 13a. The result indicates that eye-gaze tracking greatly improved the robot time. Even though the number of intermediate points detected by strategy 1 and 3 was similar, the motion planning algorithm ignored many points due to saccades, so they did not affect the results. In addition, strategy 3 improved the arrival time for the robot because of a lower detection time.

The number of robot stops was significantly higher in strategy 1 than strategy 3 (p=0.000) by 69% and strategy 2 (p=0.05) by 77%, as shown in Table 2 and Figure 13b. Although the difference in the number of intermediate points detected was insignificant between strategy 1 and strategy 3, the robot did not stop for all intermediate points. This implies that selections by strategy 3 due to saccades did not significantly affect the robot motion, thanks to the motion planning algorithm, which discarded new information received before a trajectory finished its execution.

### 4.3. Analysis of the Effect of Parameters on the Performance of the Strategies

The performance of strategy 3 depends on the values of the hand threshold parameter $\lambda_{d}$ and the eye-gaze window parameter α. Therefore, we conducted experiments to determine the effect of $\lambda_{d}$ and α on Q1, Q2, Q3, and Q4.

### 4.4. The Effect of the Hand Threshold

We experimented with different values of $\lambda_{d}$, with $\lambda_{d}=5$ cm as the baseline, compared to $\lambda_{d}=10$ cm, 15 cm, 20 cm, 25 cm, and 30 cm. The results based on a data set with 34 different trajectories are presented below.

**Figure 14:**
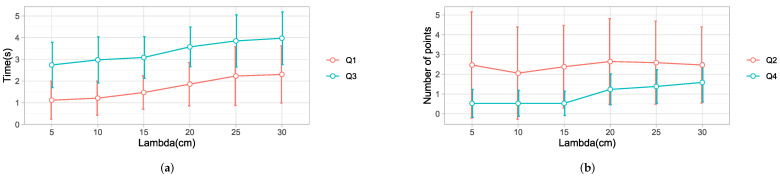
Results for each value of the hand threshold. (**a**) Q1: The time taken by the strategy to detect the desired point. Q3: The time taken by the robot to reach the desired point. (**b**) Q2: The number of intermediate points detected by the strategy. Q4: The total number of intermediate stopping points of the robot.

A one-way ANOVA model revealed a significant effect of the $\lambda_{d}$ on Q1F(5,198)=8.099, p=0.000. Post hoc comparisons using the Tukey HSD test [49] indicated that the mean time for $\lambda_{d}=5$ cm was statistically lower than that for $\lambda_{d}=25$ cm (p=0.000) by 1.11 s and $\lambda_{d}=30$ cm (p=0.000) by 1.19 s. Specifically, our results suggest that increasing the value of the threshold generally increased the time to detect the final point. A small threshold allows for the detection of the hand’s intention to move away from the current point. This leads to an early detection of the desired point by the eye gaze. However, $\lambda_{d}$ had to be greater than 20 cm to notice a significant effect. Details are shown in Table 3 and in Figure 14a.

A one-way ANOVA revealed no significant effect of $\lambda_{d}$ on Q2, with (F(5,198)=0.294, p=0.916). The results are shown in Table 3 and in Figure 14b. The difference was not significant because of the following reasons. First, a lower value of $\lambda_{d}$ triggered selection by the eye gaze, which is affected by saccades, as observed in [16], resulting in a high number of intermediate points. Increasing the threshold would reduce the saccades because the target selection is by hand. However, this would mean that the strategy would tend to behave like strategy 1, increasing the number of intermediate points. Thus, selecting the correct value for this criteria is a trade-off between selection by eye gaze and selection by user’s hand. The best balance was $\lambda_{d}=10$ cm or 15 cm.

A one-way ANOVA model revealed a significant effect of $\lambda_{d}$ on Q3 F(5,198)=7.486, p=0.000). Post hoc comparisons showed significant differences between $\lambda_{d}=5$ cm, 10 cm, 15 cm, and $\lambda_{d}=25$ cm and 30 cm. Overall, $\lambda_{d}=5$ cm took the least time, as shown in Figure 14a and Table 3. The results show that the robot took a shorter time to reach the desired point for a small threshold.

A one-way ANOVA test showed that the hand threshold had a significant effect on Q4: F(5,198=7.486),p=0.0. The results are shown in Table 3 and in Figure 14b. Post hoc comparisons revealed that $\lambda_{d}=5$ cm was significantly different from $\lambda_{d}=25$ cm and 30 cm; however, it was not significantly different from $\lambda_{d}=10$ cm and 15 cm. The results show that a larger threshold increased the number of intermediate points detected by the robot. The mean values were similar for the lower values of $\lambda_{d}=5$ cm, 10 cm, and 15 cm. Then, the slope of the graph changed with increasing values of $\lambda_{d}$. This pattern is different from the results obtained from the number of intermediate points selected by the algorithm. The algorithm selected a significant number of intermediate points for lower thresholds due to the saccades in the eye-gaze tracking. Therefore, the robot discards most of them thanks to a robust motion planning algorithm. On the contrary, as the threshold increases, the selection of points is mainly by hand. In this way, the algorithm behaves like strategy 1, which accounts for the increased number of intermediate points detected.

Overall, there was no significant difference between $\lambda_{d}=5$ cm, 10 cm, and 15 cm for all of the objectives Q1, Q2, Q3, and Q4. For this study, the best value selected was $\lambda_{d}=15$ cm, in accordance with the dimension of the environment. People hold a ball with a diameter of 7.5 cm. The tracker is placed on the top of the hand at a distance of approximately 5 cm from the palm. Therefore, the total distance from the center of the ball to the tracker was approximately 8 cm.

### 4.5. Eye-Gaze Window

Previous studies [2,10,42,43] have used different values of α, ranging from $1^{\circ }$, $30^{\circ }$, and $40^{\circ }$, which have been used for selecting objects in the gaze window. However, there was no standard value for the appropriate gaze window size. Based on a data set with 37 different trajectories, we present results of the effect of α by comparing $\alpha =5^{\circ },10^{\circ },15^{\circ },20^{\circ },25^{\circ },30^{\circ }$, and $60^{\circ }$ to the baseline $\alpha =1^{\circ }$. Normality checks were carried out and the assumptions were met.

**Figure 15:**
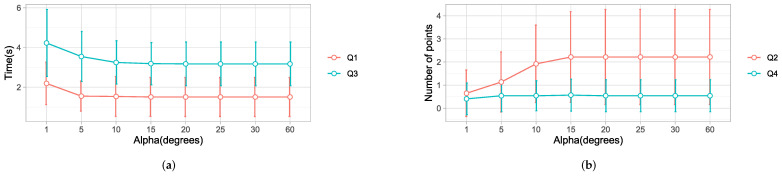
Investigating the effect of the eye-gaze threshold using different values of α. (**a**) Q1: The time taken by the strategy to detect the desired point. Q3: The time taken by the robot to reach the desired point. (**b**) Q2: The number of intermediate points detected by the strategy. Q4: The total number of intermediate stopping points of the robot.

A one-way ANOVA test showed that α had a significant change on Q1, with F(7,288)=2.230 and p=0.032, as shown in Table 4 and Figure 15a. The results from a post hoc analysis showed that $\alpha =1^{\circ }$ has a significantly longer detection time (p=0.054) than $\alpha =20^{\circ }$, $\alpha =25^{\circ }$, $\alpha =30^{\circ }$, and $\alpha =60^{\circ }$, with a difference of 0.68 s. These results show that decreasing α delayed the detection of a point because of the smaller selection. A point cannot be selected until it is within the gaze window. A threshold greater than $10^{\circ }$ would give a view cone greater than $20^{\circ }$, which would be large enough to accommodate several points in the user’s gaze direction.

A one-way ANOVA test showed that Q2 was significantly affected by α, with *F*(7, 288) = 4.237 *p* = 0.000. More specifically, a post hoc analysis showed that $\alpha =1^{\circ }$ had the lowest number of intermediate points, with a value significantly lower than $\alpha =10^{\circ }\left(p=0.003\right)$, $\alpha =15^{\circ }$, $\alpha =20^{\circ }$, $\alpha =25^{\circ }$, $\alpha =30^{\circ }$, and $\alpha =60^{\circ }\left(p=0.000\right)$. The results are shown in Table 4 and Figure 15b. This suggests that, when α was set to a value less than $10^{\circ }$, the detection of intermediate points decreased significantly. A small selection window will block out many points, whereas a large window gives room for saccades. This relationship is depicted in Figure 15b.

There were significant differences in the time taken by the robot to reach the desired point: F(7,288)=5.451, p=0.001. The time taken using $\alpha =1^{\circ }$ was significantly greater than the rest (p=0.000). These results showed that reducing α to a value $<10^{\circ }$ significantly delayed the robot. However, the difference was not noticeable between large values, as can be observed in Figure 15a.

There was no significant effect of α on Q4. Adjusting the threshold had no effect on the intermediate stops of the robot, as observed in Figure 15b. These results follow a similar pattern to the results from Q2. However, in this case, the number was lower thanks to the robust motion planning algorithm.

## 5. Discussion

This study on the development and evaluation of strategies for user motion prediction was motivated by the need to improve detection speeds and increase the response time in EHDs.

Most importantly, our solution relied on the eye-gaze direction and hand position to determine human motion intention and desired targets. We analyzed data from three participants to determine the time taken by each strategy to detect the desired point, the number of intermediate points detected, the time taken by the robot to reach the final point, and the total number of intermediate stopping points of the robot. Strategy 3 gave the best detection time, robot time, and fewer robot stops. These results showed that the eye gaze significantly improved the response time while minimizing the number of robot stops. Our results were coherent with the literature on hand-eye coordination and target selection, which has identified that humans typically fix their gaze in the direction of the target, slightly before or after the hand begins to move, as shown in Figure 9.

The results suggest that visual behaviour for target selection with a haptic system is similar to behaviour when carrying out the task with hands in everyday life. Thus, the proposed system should work for people with motor impairments.

The prediction strategy based on the eye-gaze direction demonstrated a pattern to detect more intermediate points because of the saccadic movements. To minimize this behaviour, recent studies [15] in which the gaze direction is used to predict human intention utilized gaze attention models. In such models, they wait for a window period ranging from 200 ms to 4 s when the gaze is fixated on an object to validate it as a target. Such models affect robot arrival times and are applicable for large objects. In our case, the balls are not big. Thus, we used a threshold on the hand to limit the selection of the next point. The detection by the eye gaze was cut off when the point-to-hand distance was less than a threshold. In addition, the path-planning algorithm of the robot was designed to complete a trajectory before starting a new one. Thus, rapid trajectory changes due to saccades were always discarded. This implies that our model can be used for both small and large objects, as long as a suitable threshold on the hand is selected.

In this study, the hand threshold plays a vital role in detecting human motion intention. In studies where the nearest point to the hand method is used [2,18], the target was detected whenever the hand had crossed half the distance between any two points. However, when coupled with the eye gaze, a hand threshold was used to detect the user’s intention to move to another point. In this way, the hand-to-point was below the threshold, and the user intention was interpreted as a desire to remain at a point. Therefore, the robot remained stationed at the point. The threshold also served to restrict the detection of a new point. Thus a threshold plays a significant role in determining the detection time of the target and the intermediate points. Thus, it affects the robot arrival time and the intermediate points detected along the robot trajectory. We experimented with different threshold values on the hand to determine a suitable threshold value. The analysis revealed that a lower threshold was associated with a faster detection time. We attribute this to the fact that a lower threshold value indicated an earlier detection of the intention by the user to move to another point.

Due to the lack of clear agreement on the standard size of the gaze view window in studies investigating eye gaze and hand coordination patterns [2,10,42,43], we examined the effect of the threshold on the eye gaze. Our results showed that a view angle greater than $20^{\circ }$, as used in strategy 3, had similar results for all of the research questions Q1, Q2, Q3, and Q4. However, a reduced threshold $\leq 10^{\circ }$ was associated with a significantly increased time for detection, but reduced the number of intermediate points. A small threshold implied that a few points would be selected at a time. Thus, it would take longer to have a valid selection, which greatly increased the time to detect the desired point and consequently delayed the robot. From the analysis, we discovered that the gaze fixation model, as used in [2], increased the detection time, hence delaying the robot.

Previous studies [16,50] pointed out that the visual gaze is full of rapid eye movements between fixed points (saccades), and it was the primary reason why gaze fixation was the widely used approach. However, we do not use gaze fixation because it takes longer to detect a point. In addition, it is unsuitable for smaller objects. Instead, we used a threshold on the hand to restrict robot motion when a point lies within the stated threshold. We select the best point depending on the angle α and not the visual ray directly. By combining the hand threshold and a good motion-planning algorithm, saccadic movements do not significantly affect the robot motion when a large threshold on the eye gaze is used. Thus, our approach is robust to saccades and highly responsive.

Our findings show that prediction based on the eye gaze improved the response time for the robot. However, optimizing the detection time from human predictions comes at the cost of increasing the intermediate points detected. We observed this through an analysis of the threshold values on the hand. A lower value resulted in a good detection time and a higher value of intermediate points detected. Thus, a compromise has to be made to improve the detection time and reduce the detection of the intermediate points. Therefore, we recommend finding a suitable threshold on the hand and the eye-gaze window to suit the task.

In addition, our system only uses positions in a 3D space; it would be good to extend the interaction to 6-DOF to study the implications of prediction and robot time in haptic rendering systems where positions and orientations of virtual objects are essential. Although VR hand-eye coordination significantly improved the detection time, we observed that participants spent some time searching for a target. Therefore, further research is needed to minimize the time spent searching for the next target to increase the user’s performance and the eye-hand coordination training system. Our work was preliminary on the proof of concept with a few healthy participants. It would be essential to evaluate the haptic system with many people with motor disabilities.

Eye-gaze detection to predict targets for haptic devices is a promising solution to improve intention detection and robot response. However, due to saccades during decision making and target search, additional studies are needed on methods to process gaze data.

## 6. Conclusions

Haptic systems enable physical user interaction with a virtual world by automatically recreating virtual scenes for dynamic interactions through haptic rendering. However, speed constraints present a challenge for the real-time interaction of such systems. We have addressed this problem through motion prediction using eye-gaze direction and the user’s hand. This study developed motion prediction strategies in a virtual environment for reaching tasks. Based on data from three participants, our study confirms the principle that the eye gaze precedes hand movement for reaching tasks. Furthermore, our results confirm that the strategy using eye-gaze-based prediction significantly reduced the detection of the desired point and reduced intermediate points. This significantly improved the robot response, with fewer intermediate robot stops. More specifically, our approach showed better results than the state-of-the-art, which relies on gaze fixation. Therefore, this approach may be helpful to communities using haptic systems for upper extremity rehabilitation training and tasks for rapid prototyping in industrial design [51] to improve the response time and device speed.

## Figures and Tables

**Table 1:** Strategy results for the user’s hand trajectory from $P_{1}$ to $P_{16}$.

Strategy	Q1	Q2	Q3	Q4
1	4.86	2.0	6.08	2.0
2	5.34	0.0	7.87	0.0
3	4.12	1.0	5.35	1.0

**Table 2:** Mean and standard deviation of Q1, Q2, Q3, and Q4 for different strategies.

	Q1		Q2		Q3		Q4	
**Strategy**	**Mean**	**SD**	**Mean**	**SD**	**Mean**	**SD**	**Mean**	**SD**
1	2.47	1.34	2.63	1.62	4.18	1.36	1.84	1.13
2	2.82	1.36	0.50	0.86	4.89	2.06	0.42	0.68
3	1.54	0.99	2.32	2.12	3.23	1.14	0.58	0.72

**Table 3:** Analysis of Q1, Q2, Q3, and Q4 for different threshold values.

$\lambda_{d}$	Q1		Q2		Q3		Q4	
	**M**	**SD**	**M**	**SD**	**M**	**SD**	**M**	**SD**
5 cm	1.12	0.88	2.47	2.69	2.74	1.04	0.53	0.71
10 cm	1.22	0.79	2.06	2.33	2.98	1.06	0.53	0.66
15 cm	1.48	0.77	2.38	2.09	3.09	0.96	0.53	0.61
20 cm	1.86	1.00	2.65	2.17	3.58	0.92	1.24	0.78
25 cm	2.23	1.36	2.59	2.11	3.85	1.20	1.38	0.85
30 cm	2.31	1.32	2.47	1.93	3.97	1.21	1.59	0.99

**Table 4:** Mean and standard deviation of Q1, Q2, Q3, and Q4 for different α.

α	Q1		Q2		Q3		Q4	
	**M**	**SD**	**M**	**SD**	**M**	**SD**	**M**	**SD**
$1^{\circ }$	2.19	1.07	0.65	1.01	4.23	1.69	0.41	0.69
$5^{\circ }$	1.55	0.76	1.14	1.29	3.55	1.26	0.54	0.69
$10^{\circ }$	1.53	1.01	1.92	1.67	3.25	1.09	0.54	0.65
$15^{\circ }$	1.51	0.98	2.22	1.96	3.19	1.06	0.57	0.69
$20^{\circ }$	1.51	0.98	2.22	2.06	3.18	1.10	0.54	0.69
$25^{\circ }$	1.51	0.98	2.22	2.06	3.17	1.10	0.54	0.69
$30^{\circ }$	1.51	0.98	2.22	2.06	3.17	1.10	0.54	0.69
$60^{\circ }$	1.51	0.98	2.22	2.06	3.17	1.10	0.54	0.69

## Supplementary Materials

The following supporting information can be downloaded at: https://www.mdpi.com/article/10.3390/s22052040/s1, A participant performing the experimental exercise.
